# Plasmin, Immunity, and Surgical Site Infection

**DOI:** 10.3390/jcm10102070

**Published:** 2021-05-12

**Authors:** Stuart Hastings, Paul S. Myles, Robert L. Medcalf

**Affiliations:** 1 Department of Anaesthesiology and Perioperative Medicine, Alfred Hospital, Melbourne, VIC 3004, Australia; p.myles@alfred.org.au; 2 Department of Anaesthesiology and Perioperative Medicine, Monash University, Melbourne, VIC 3004, Australia; 3 Australian Centre for Blood Diseases, Monash University, Melbourne, VIC 3004, Australia; robert.medcalf@monash.edu

**Keywords:** antifibrinolytics, fibrinolysis, infection, sepsis, surgery, tranexamic acid

## Abstract

SSI are a universal economic burden and increase individual patient morbidity and mortality. While antibiotic prophylaxis is the primary preventative intervention, these agents are not themselves benign and may be less effective in the context of emerging antibiotic resistant organisms. Exploration of novel therapies as an adjunct to antimicrobials is warranted. Plasmin and the plasminogen activating system has a complex role in immune function. The immunothrombotic role of plasmin is densely interwoven with the coagulation system and has a multitude of effects on the immune system constituents, which may not always be beneficial. Tranexamic acid is an antifibrinolytic agent which inhibits the conversion of plasminogen to plasmin. Clinical trials have demonstrated a reduction in surgical site infection in TXA exposed patients, however the mechanism and magnitude of this benefit is incompletely understood. This effect may be through the reduction of local wound haematoma, decreased allogenic blood transfusion or a direct immunomodulatory effect. Large scale randomised clinical trial are currently being undertaken to better explain this association. Importantly, TXA is a safe and widely available pharmacological agent which may have a role in the reduction of SSI.

Current prevention of surgical site infection (SSI) largely relies upon strict hygiene practices during and after surgery, and targeted antibiotic prophylaxis. More than 313 million people undergo surgery worldwide each year, with SSIs one of the most common and economically burdensome postoperative complications [1]. The reported incidence is likely underestimated [2]. However, SSI is thought to occur in 0.5% [3] to 23% [4] of surgeries, depending on the type of intervention, with a heightened prevalence amongst countries with a low Human Development Index. At an individual patient level, SSI results in prolonged hospital stay, readmission, reoperation, and a 2- to 11-fold increased risk of mortality [2,5], while attributed pain and anxiety negatively impacts health-related quality of life [5]. The economic implications of SSI are stark. In the United States, each case increases hospital costs by more than $20,000 per admission, with $700 million annually being attributed to readmission, for this complication alone [2]. This global burden means that effective complementary, adjunctive, or alternatives to antibiotics that can be universally instituted are required, to reduce the global impact of SSIs.

The pathogenesis of SSIs relates to the complex interplay between the magnitude of wound inoculum, organism pathogenicity, and efficiency and strength of the patient immune response. Prophylactic antimicrobial therapy with an efficacious agent of appropriate spectrum and time to surgical intervention was repeatedly shown to reduce SSI, and constitutes the primary pharmacological strategy to reduce the surgical site infection risk endorsed by multiple international society-based guidelines [6,7,8]. This strategy aims to reduce the burden of inoculum by stifling microorganism proliferation at the surgical site. However, it is increasingly appreciated that antimicrobial therapy is not benign and might be ineffective in some instances.

The emerging international problem of antibiotic resistance threatens the effectiveness of a reliance on antibiotic prophylaxis and treatment of SSIs. A recent study investigating SSI in gastrointestinal surgery found greater than one-fifth of organisms contributing to an SSI might be resistant to the prophylactic agent, with the prevalence of resistant organisms inversely proportional to a country’s Human Development Index. SSIs [4] by themselves are likely to further contribute to antibiotic resistance [9].

The 6th National Audit Project (NAP-6) in the United Kingdom identified antibiotics as the likely culprit in 46% of cases of perioperative anaphylaxis, with an overall incidence of 4 per 100,000 administrations [10]. This figure was higher for specific agents most commonly utilized as surgical prophylaxis, with an anaphylaxis incidence of 8.7 per 100,000 for co-amoxiclav and 16.7 per 100,000 for teicoplanin, the agent most commonly selected as a penicillin alternative [10].

Immunological impairment secondary to disease processes, comorbidities, or pharmacological agents were consistently identified as a patient-centered risk factors for SSI. Minimizing the impact on native defenses is a suggested practice, as part of a multifaceted strategy to reduce SSI. Advocated means [6,7,8] include perioperative reduction or withdrawal of pharmacological agents invoking immune suppression, such as glucocorticoids or tumor necrosis factor inhibitors [11,12], optimization of comorbidities, and limitation of environmental influences like hypothermia, which might retard the immune function. The use of pharmacological agents to enhance the immune system performance is not featured.

## 1. A Role for Plasmin as an Immune Modulator

One novel means to reduce SSIs that has come into light is targeting the plasminogen activating (“fibrinolytic”) system. This system orchestrates the production of the serine protease plasmin, from its zymogenic precursor, plasminogen, and is well-known for its role in fibrin clot dissolution. However, it is now becoming well-appreciated that this pathway impacts various other processes that are unrelated to fibrin removal [13,14]. Part of this relates to the close relationship between the fibrinolysis, coagulation, and the complement pathways, as the enzymes and regulatory molecules of all three systems co-evolved. With these close ancestral ties, it is not at all surprising that proteases of one pathway can cross-activate another. Indeed, cross-activation of the coagulation system by the complement pathway is well described [15]. Similarly, plasmin, can activate the key complement factors, C5 and C3, while on the other hand, it can itself be inhibited by the C1-inhibitor, thereby providing a natural means to regulate this process. Plasmin was also reported nearly 70 years ago to effectively inactivate the complement pathway [16] and this was confirmed in recent times [17]. Hence, plasmin can be both pro- and anti-inflammatory, which might depend on the timing and the load of the inflammatory challenge. Opposing actions of plasmin were also reported in animal models of sepsis [18]. Plasmin can also activate key members of the contact pathway, including Factor XII and pre-kallikrein. This is bi/tri-directional, as these key proteases of the contact pathway also activate plasminogen into plasmin [19,20]. One prime purpose of this cross-activation is to amplify the host response to various immune and inflammatory challenges, but this is not always beneficial if over-stimulated, as in the event of anaphylaxis [21,22].

In addition to this, plasmin itself is a broad spectrum serine protease with numerous substrates. Although fibrin is arguably its most renowned substrate, plasmin is a critically important enzyme that can process many proteins from their inactive (“pro”) to active (“mature”) forms, including transforming growth factor—beta TGF- [23], a neurotrophic agent brain-derived neurotropic factor [24], and other proteases like the matrix metalloproteinases [25].

Enhanced understanding of the extensive role of plasmin and the plasminogen activating system solidified the concept of an intertwining of the hemostatic and immunological systems [26], and the particular interest in the role of plasminogen activation increased in recent years [27,28,29]. The immunological and inflammatory effects of plasmin are indeed pronounced [30]. Plasmin can directly increase macrophage and dendritic cell phagocytosis in a manner that represses immune cell activation [31,32], at least in part by promoting large increases in the release of transforming growth factor (TGF) [31]. Plasmin can also be either pro- or anti-inflammatory depending on the timing of immune challenge [18] and can directly promote cytokine release [33,34,35]. Many of these actions of plasmin occur on the cell surface of various immune cells. Indeed, at least 12 receptors exist for plasminogen, many of which bind to plasminogen via lysine residue interactions. Plasmin, once formed on the cell surface, can cleave other protein substrates, some of which can promote cell activation that in turn can modulate cell behavior [30]. In models of traumatic brain injury, plasmin formation can restrict movement of innate immune cells to regional lymph nodes and can alter the cell surface expression pattern of various activation markers [30,36,37,38].

Consistent with the role of plasmin on inflammatory and immune processes, the plasminogen activation process is also a key modulator of wound healing. Mice deficient in plasminogen show markedly reduced capacity to heal full thickness cutaneous wounds [39,40] and this was further emphasized in models of tympanic membrane repair [41] and in radiation-induced injury [42,43]. In addition to directing trafficking of immune cells and promoting phagocytosis, conventional roles of plasmin also come into play via its ability to efficiently remove misfolded proteins and necrotic tissue, in a manner indistinguishable from fibrin removal [44,45], which is also critical in the tissue repair process.

While mammals harnessed the plasminogen activating system for these critical process, infectious organisms themselves developed countermeasures where they can harness host plasminogen to generate plasmin, to avoid host immunity. Most, if not all bacteria, as well as many fungi and protozoan and helminth parasites, have the capacity to produce their own fibrinolytic agents, or (more commonly) can capture host plasminogen on their surface [46]. It is also interesting to note that some strains of streptococci produce a fibrinolytic protease (streptokinase) that can only activate human plasminogen, but not plasminogen from any other species [47,48]. The resulting plasmin can release the pathogens from fibrin barriers formed to entrap them, while at the same time inactivating complement and suppressing other host immune parameters.

## 2. Tranexamic Acid as an Agent to Mitigate Surgical Site Infection

With this expanding interest in plasminogen activation and innate immunity, the therapeutic modulation of plasmin formation is likely to influence immune defense mechanisms of the host as well as infectious pathogens. Tranexamic acid (TXA) is a potent anti-fibrinolytic agent that was used for decades to reduce bleeding. However, detailed understanding of the particulars relating to timing and the extent of plasmin’s immunological impact are incompletely elucidated. Given TXA’s potency at inhibiting plasmin formation, it is well-placed to be considered as a novel agent with the potential to alter the native immune response and favorably influence the incidence of wound infection, however, the translation to clinical practice was not comprehensively demonstrated.

**Figure 1:**
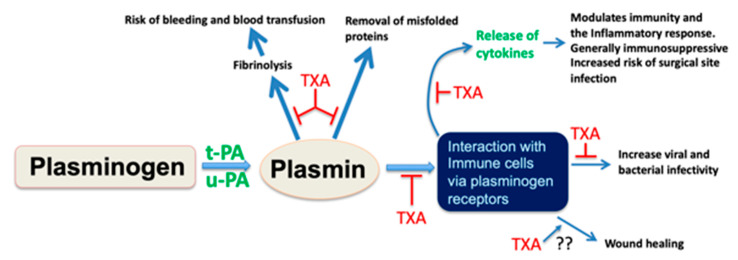
The generation of plasmin from its precursor, plasminogen is achieved by the plasminogen activators, tissue-type plasminogen activator, and urokinase (tPA and uPA, respectively). Plasmin once formed can cleave fibrin and other misfolded proteins. Excessive plasmin formation can result in hyperfibrinolysis, which increases the risk of bleeding and blood transfusion needs. Plasmin can also be formed on cell surfaces, including immune cells, via specific plasminogen receptors. This can result in the release of various cytokines with subsequent effects on cell behavior, inflammation, and immunity. Tranexamic acid (TXA) blocks lysine-dependent interactions and therefore inhibits binding of plasminogen to the surface of fibrin and misfolded proteins. The protection and stabilization of fibrin by TXA also reduces bleeding risk and transfusion requirements. Plasminogen receptors located on the surface of immune cells also contain C-terminal lysine residues that are important for plasminogen binding and subsequent cell activation. Hence, TXA can inhibit this interaction and therefore block downstream signaling events and cytokine release. It remains to be determined whether TXA blocks wound healing, although the transient administration of TXA in most clinical settings makes this unlikely.

Tranexamic acid was reported to be an inhibitor of fibrinolysis by Shosuke and Okamoto in the early 1960s [49], and was initially implemented in clinical practice as a pharmacological agent for the management of menorrhagia and heritable bleeding diatheses. TXA is a lysine analogue that competes with plasminogen to bind to the exposed lysine residues on the fibrin surface. Hence, when in excess, the plasminogen binding sites are occupied by TXA and, therefore, are unavailable to bind to the lysine binding sites on fibrin, thereby, sparing fibrin from plasmin-mediated destruction. The mechanism of action of TXA, therefore, is fundamentally different from aprotinin, which is a direct plasmin inhibitor (Figure 1).

The potency and success of TXA at reducing pathological bleeding saw the exploration of this benefit in the perioperative setting. A multitude of randomized and observational trials across diverse surgical populations, including cardiac, orthopedic, neurosurgical, intraabdominal and ear, nose, and throat, with variable bleeding and thrombosis risk demonstrated tranexamic acid as a safe and efficacious agent for this indication [50]. A distinct survival advantage was seen in a number of these groups [50,51,52]. Evidence of a mortality benefit with no heightened risk of thromboembolic events was preserved in a large, pooled analysis, comprising a diverse range of surgical sub-groups [53].

The decrease in surgical bleeding in response to TXA administration might have the potential to mitigate SSI by both local and systemic mechanisms. TXA reduces the incidence of wound hematoma [54,55], which serves as a nidus for wound infection [56]. Reduction in the volume of this nutrient-rich medium might limit the pathogen burden and permit local control by the innate immune system.

The minimization or elimination of allogenic blood transfusions in the surgical setting is a highly desirable outcome. Receipt of allogenic blood was repeatedly shown to be strongly associated with postoperative mortality, ischemic complications including stroke, myocardial infarction, and renal impairment, prolonged hospital length of stay, and SSI [57,58,59,60,61,62]. Transfusion-related immunomodulation (TRIM) is an umbrella definition describing the phenomena of immune dysfunction attributed to the proinflammatory and immunosuppressive effects of packed red blood cell transfusion. The mechanism of this phenomenon is diverse and multifactorial and might relate to white blood cell priming and enhanced chemotaxis, activation of monocytes and macrophages, impaired NK cell function, defective antigen presentation and cytokine release [63]. While routine leukoreduction serves to reduce the immunosuppressive constituents derived from white blood cells, the cytokine content and other soluble mediators remains sufficient to have a deleterious effect via Treg cell activation [64]. A postulated mechanistic effect of TXA on a favorable outcome with respect to SSI is the reduction of the allogenic red-cell-induced immune suppression, as opposed to any direct effect on immune function by TXA itself [65]. However, enhanced understanding of the diverse immunological role of plasmin and the plasminogen activating system challenges this explanation.

The Aspirin and Tranexamic Acid for Coronary Artery Surgery (ATACAS) trial was a multicenter double-blind randomized controlled trial investigating the use of TXA in patients undergoing coronary artery bypass surgery, at heightened risk for major complications [66]. A planned sub-study of patients from this population evaluated the immunomodulatory effects of TXA and its effect on reducing SSI [67]. At a biochemical level, immune enhancement was observed, with an alteration in peripheral blood myeloid and lymphoid cell immune activation marker expression, and reduced levels of pro-inflammatory cytokine production. This coincided with a significant reduction in SSI (14.3% vs. 20.6%, *p* = 0.041) independent of the difference in allogenic blood product administration.

Subset analysis of the diabetic cohort revealed a potential confounding effect, whereby TXA did not reduce the rate of wound infection in patients with diabetes who were TXA-naïve (35% vs. 35%; *p* = 0.99). This might be explained by glycosylation of plasminogen, preventing its activation, and from an increase in plasma antifibrinolytic proteins [68,69]. TXA mitigated the allogenic transfusion requirement in the diabetic cohort, though less effectively, with an RR reduction of only 28% vs. 40% for non-diabetics (*p* for interaction 0.012). These preliminary findings suggest TXA could be at least partially refractory in the diabetic population.

The mechanistic effect by which TXA might reduce SSI is potentially related to its direct immunomodulatory effect, by limiting the deleterious effects of plasmin. This might be independent of a reduction in the immunological insult associated with autologous blood transfusion. As such, TXA might have an extended role in the surgical setting beyond its established use in blood-conserving strategies, with universal application across a diverse range of interventions independent of bleeding risk.

As yet, large pragmatic randomized controlled trials addressing the impact of TXA for the purpose of mitigating SSI are lacking. Enrolment in the Tranexamic Acid to Reduce Infection after Gastrointestinal Surgery (TRIGS) trial (ClinicalTrials.gov Identifier: NCT04192435) is in progress. This large, multicenter trial of 3300 patients is a clinical and biochemical investigation into the effects of TXA, with respect to the effect on SSI and the temporal modulation of the immunological and inflammatory response to this surgical insult. The hypothesis is in keeping with the plasmin hypothesis on immune function, and a favorable outcome with a reduction in the rate of SSI is anticipated.

## 3. Conclusions

The considerable economic impact of SSI and the heightened risk for patient morbidity and mortality means investment in the search for mitigating strategies is warranted. While there is unlikely to be a single intervention that comprehensively minimizes this eventuality, a cheap and universally available pharmacological agent that enhances the native host immune response, which is at minimal risk of causing individual patient complications, and which does not contribute to the global burden of antibiotic resistance would be a novel and clinically meaningful intervention. The suitability of TXA to fulfil this requirement remains to be answered. Additional understanding of its immunomodulatory effects at a laboratory level and translational to the clinical setting will further define its role.
